# Association of gut microbiota and regulatory T cells with vaccine immunogenicity in HIV-exposed uninfected and HIV-unexposed infants: a longitudinal cohort study

**DOI:** 10.1016/j.ebiom.2026.106500

**Published:** 2026-10-01

**Authors:** Michael J. Johnson, Daniel N. Frank, Adriana Tovar Salazar, Marta C. Nunes, Shabir A. Madhi, Adriana Weinberg

**Affiliations:** a Department of Pediatrics, University of Colorado Anschutz Medical Campus, Aurora, CO, USA; b Department of Medicine, University of Colorado Anschutz Medical Campus, Aurora, CO, USA; c Faculty of Health Sciences, South African Medical Research Council Vaccines and Infectious Diseases Analytics Research Unit, Department of Science and Technology/National Research Foundation South African Research Chair Initiative in Vaccine Preventable Diseases, University of the Witwatersrand, Johannesburg, South Africa; d Faculty of Health Sciences, African Leadership in Vaccinology Expertise, University of the Witwatersrand, Johannesburg, South Africa

**Keywords:** Microbiome, HIV-exposed uninfected, Treg, Vaccines, Immunity

## Abstract

**Background:**

Responses to vaccines reflect the functionality of the immune system. We measured BCG, tetanus and measles vaccine responses in HIV-exposed uninfected (HEU) and HIV-unexposed (HUU) infants to identify factors associated with HEU immunologic defects.

**Methods:**

We assessed cell-mediated immunity (CMI) by FluoroSpot, antibodies by ELISA, regulatory and immunologic checkpoint inhibitor-expressing T cells (Tregs/Ticis) by flow cytometry, and gut microbiota by 16S rRNA gene sequencing. CMI and antibody responses were the primary outcome measures. Associations with Tregs/Ticis and gut microbiome parameters were identified using the hurdle model, Spearman correlations and enrichment analyses.

**Findings:**

Among 123 HEUs and 117 HUUs, HEUs tended to have lower BCG-CMI than HUUs without reaching statistical significance (p = 0.07) and similar tetanus-CMI, measles-CMI, and measles-antibodies. Multiple Treg and Tici subsets had significant or marginal negative effects on CMI (p = 0.003–0.10). Treg/Tici inclusion in the hurdle model decreased the statistical significance of the negative effect of HEU status on BCG-CMI suggesting that Tregs/Ticis may have contributed to the difference between HEUs and HUUs. Enrichment of the gut microbiota in Actinobacteria and Firmicutes was significantly associated with low vaccine immunogenicity (p = 0.002–0.04), and Bacteroidetes and Proteobacteria with high immunogenicity (p < 0.0001–0.10). The Firmicute *Murdochiella sp.* had a higher negative effect size on BCG-CMI in HEUs than HUUs (p < 0.0001). High microbiome diversity negatively correlated with CMI responses (p = 0.002–0.03), with higher effect sizes in HEUs than HUUs (p = 0.047).

**Interpretation:**

The frequency of Tregs/Ticis and the composition of the gut microbiome are associated with immune responses to vaccines and may contribute to decreased responses in HEUs. The internally consistent association between the enrichment of gut microbiota in specific phyla and the immunogenicity of several vaccines suggests the possibility of designing mitigating interventions that target the gut microbiome.

**Funding:**

National Institute of Allergy and Infectious Diseases and Eunice Kennedy Shriver National Institute of Child Health and Human Development, National Institutes of Health.

**Research in context**

**Evidence before this study**

HIV-exposed uninfected (HEU) infants have higher infectious morbidity and mortality than HIV-unexposed (HUU) infants. The increased severity of infections in HEU infants has been ascribed to decreased protective immune responses. Although multiple immunologic differences between HEU and HUU infants have been described, the mechanisms underlying these differences have not been elucidated, hindering the development of mitigating interventions. HEU and HUU infants also differ with respect to their gut microbiome, which plays a paramount role in the development of the infant immune system. However, a clear association between decreased immune responses in HEU infants and the gut microbiome composition had not been established. Previous studies investigated the association of the gut microbiome composition with immune responses to vaccines in healthy infants. The results were largely discrepant, but at least two groups found that the gut microbiome composition around the time of vaccination was associated with the immunogenicity of vaccines, and/or that high abundance of genera belonging to the phylum Bacteroidetes were associated with high responses to vaccines, and/or that the abundance of *Bifidobacterium spp.* positively correlated with the magnitude of responses to vaccines.

**Added value of this study**

In this study, we used immune responses to vaccines as a surrogate marker of the functionality of the HEU and HUU immune systems. We found that immune responses to BCG were marginally lower in HEU than HUU infants, consistent with an immunologic defect in HEU. Responses to BCG, tetanus, and measles vaccines were negatively associated with the abundance of genera belonging to the Actinobacteria and Firmicute phyla at the time of immunization, while Proteobacteria and Bacteroidetes were positively associated with immune responses to vaccines in both HEU and HUU infants. Moreover, most associations with phyla enrichment applied to responses to ≥2 vaccines. Features of the gut microbiota associated with lower responses to vaccines had a more pronounced effect in HEU than HUU infants.

**Implications of all the available evidence**

Our study adds to the body of evidence associating the gut microbiome composition with infant immune responses against bacterial and viral antigens. We identified new potential targets for mitigating interventions aimed at increasing infant immune responses to vaccines and possibly to other microorganisms. The potential targets include increasing the abundance of Proteobacteria and Bacteroidetes and decreasing the abundance of Actinobacteria in the gut microbiome.

## Introduction

Due to extraordinary advances in the prevention of HIV vertical transmission, the number of HIV-exposed uninfected infants (HEUs) has been constantly increasing surpassing two million births/year. HEUs have higher infectious morbidity, including increased incidence of hospitalizations and deaths compared with HIV-unexposed infants (HUUs) during the first 6–24 months of life.^1^, ^2^, ^3^ The exact mechanism underlying this observation is not known, but it is likely due to immunologic dysfunction.

Multiple abnormalities in T cell and antigen presenting cell (APC) phenotypes and function were identified in HEUs, but their contribution to their decreased immune defenses against infections has not been elucidated.^4^, ^5^, ^6^, ^7^, ^8^, ^9^, ^10^, ^11^, ^12^, ^13^, ^14^, ^15^, ^16^, ^17^ Studies of immune responses to vaccines provided additional information on the functionality of the adaptive immune system in HEUs. These studies generally reported similar or increased antibody responses in HEUs than HUUs and decreased cell-mediated immunity (CMI).^9^, ^10^, ^11^, ^12^, ^13^, ^14^, ^15^^,^^18^ Understanding the mechanisms underlying the differences in immune responses to vaccines between HEUs and HUUs may reveal areas that could be targeted by mitigating interventions.

We previously showed that compared to HUUs, HEUs have increased proportions of regulatory T cells (Tregs) and T cells expressing immunologic checkpoint inhibitors (Ticis), which correlated with differences in the gut microbiome between HEU and HUU mother-infant dyads and with maternal HIV infection characteristics at birth.^19^ Tregs and Ticis have broad-spectrum modulatory activity and may contribute to the increased susceptibility to severe infections.^20^, ^21^, ^22^, ^23^ Moreover, the gut microbiome plays a paramount role in the development of the immune system in children.^24^, ^25^, ^26^, ^27^ We reasoned that the differences in Treg and Tici frequencies and in the gut microbiome composition between HEUs and HUUs may contribute to the decreased immune responses of HEUs. Thus, studying the association of the microbiome composition and the frequencies of Tregs and Ticis with immune responses to vaccines in HEUs and HUUs was likely to reveal the role played by these parameters on infant immune responses.

The goal of this study was to identify differences in CMI and antibody responses to Bacille Calmette-Guerin (BCG), tetanus toxoid, and measles vaccines in South African HEUs and HUUs and evaluate the relationship of the immunogenicity of the vaccines with the abundance of infant Tregs and Ticis, and with the maternal and infant gut microbiome composition.

## Methods

### Study design

The inclusion criteria for all women (the mothers of the infants in this study) were ability to sign informed consent, singleton term gestation, planned vaginal delivery, intent to breastfeed and no antibiotics during the last month prior to delivery. Additional inclusion criteria for women with HIV were antiretroviral therapy during pregnancy. Of 3515 women, including 1074 with and 2441 without HIV, 123 women with HIV and 117 without HIV met eligibility criteria and agreed to participate in the study. After providing informed consent, maternal and infant demographic and clinical characteristics were abstracted from medical records. Maternal blood was obtained at delivery for CD4+ T-cell and HIV plasma RNA. Infant cord blood and peripheral blood at 6, 28, and 62 weeks of life was obtained for immunologic studies. Maternal rectal swabs for microbiome analysis were collected at delivery and 62 weeks postpartum and infant rectal swabs at 6, 28, and 62 weeks of life. Infants who received antibiotic therapy other than cotrimoxazole in HEUs and mothers with any antibiotic therapy within one month prior to the rectal swab collection, were excluded from the microbiome analysis.

### Flow cytometric Treg/Tici characterization

This was performed on *cryopreserved* PBMC using two panels (A and B) as previously described.^19^ Briefly, cells in both panels were stained with Zombie yellow viability die (Biolegend, cat# 423104). In panel A, we used mAbs anti-CD25 FITC (BioLegend, cat# 356106, RRID: AB_2561863), -Lag3 PE (Invitrogen, cat# 12-2239-42, RRID: AB_2572597), -CTLA4 PE-CF594 (BD Biosciences, cat# 562742, RRID: AB_2737761), -CD4 PerCP-Cy5.5 (BD Biosciences, cat# 560650, RRID: AB_1727476), and -CD3 Ax700 (BD Biosciences, cat# 557943, RRID: AB_396952) for extracellular staining. After fixation and permeabilization with Foxp3/Transcription Factor Staining Buffer Set (eBioscience, cat# 00-5523-00), intracellular staining used mAbs anti-FoxP3 Ax647 (BD Biosciences, cat# 561184, RRID: AB_10584328), -Granzyme B (GranzB) APC-fire750 (BioLegend, cat# 372210, RRID: AB_2728377), -IL-10 BV421 (BioLegend, cat# 501422, RRID: AB_2632952) and -TGFβ PE-Cy7 (BioLegend, cat# 349610, RRID: AB_10679193). In panel B, we used mAbs anti-CD4 FITC (BioLegend, cat# 317408, RRID: AB_571951), -CD3 PE-CF594 (BD Biosciences, cat# 562280, RRID: AB_11153674), GITR PerCP-Cy5.5 (BioLegend, cat# 371218, RRID: AB_2687166), -TNFR2 PE-Cy7 (BioLegend, cat# 358412, RRID: AB_2564396), -CD39 Ax700 (R&D Systems, cat# FAB4397N-100, RRID: AB_3650207), -PD1 APC-Cy7 (BioLegend, cat# 367416, RRID: AB_2616744) and -TIGIT BV421 (BioLegend, cat# 372710, RRID: AB_2632925) for extracellular staining, - FoxP3 Ax647 (BD Biosciences, cat# 561184, RRID: AB_10584328) and -IL-35 PE (BioLegend, cat# 360904, RRID: AB_2562880) for intracellular staining.

All antibodies were commercially obtained and validated by the manufacturer. Before the use in study assays, antibodies were titrated using human PBMC to verify accuracy and determine the optimal working concentration. Single-stained compensation controls were used to calculate fluorescence compensation, and fluorescence-minus-one controls were used to establish gates. Reproducibility was ensured by the inclusion in each run of PBMC from a study-specific anonymous leucopak control derived from the same healthy donor. The leucopak consisted of residual cells from platelet donations performed at the Children's Colorado Blood Donor Center. Data were analyzed using a Galios instrument (Beckman Coulter) and Flow Jo software v10 (Becton Dickinson). Figure S1 shows the gating strategy.

### FluoroSpot assays

These were performed on cryopreserved PBMCs using Mabtech FluoroSpot Plus: Human IFNγ/IL-2 (Cat# FSP-0102-10) as previously described.^28^ Thawed PBMCs with viability ≥70% were resuspended in RPMI 1640 (Corning) with 10% fetal bovine serum (FBS; Gemini Bio-Products), 1% glutamine (Gemini Bio-Products), 1% penicillin-streptomycin (Gemini Bio-Products), and HEPES buffer (Corning). After overnight resting at 37°C in a humidified 5% CO_2_ atmosphere, cells with viability ≥70% were stimulated at 250,000 cells/well in duplicate wells with 10,000 PFU/well of measles viral antigen (ATCC VR-24™), 2.5 μg/ml tetanus toxoid (Millipore Sigma Catalog # 582231), 5 μg/ml of *Mycobacterium tuberculosis* cell membrane fraction (NR-14832 of strain CDC1551; BEI Resources, NIAID, NIH),^29^ medium negative control and PHA positive control. Reproducibility was ensured by the inclusion in each run of PBMC from a study-specific leucopak control. After 48 h of incubation at 37°C in a humidified 5% CO_2_ atmosphere, plates were stained as per manufacturer's instructions and spots were counted using a CTL ImmunoSpot analyzer (Cellular Technology Limited, Ohio US). Results are reported as the average spot forming cells (SFC) per one million PBMC in antigen-stimulated wells after subtraction of SFC in unstimulated control wells.

### Measles-specific antibodies

IgG antibodies against measles were measured in plasma using a commercial indirect ELISA kit (Enzygnost, Siemens Health Care Diagnostic Products, Marburg, Germany, Catalog # OWLN15) as per manufacturer's instructions. Briefly, diluted samples were incubated in measles-virus antigen and control-antigen wells, followed by incubation with peroxidase-conjugated anti-human IgG and colorimetric detection with tetramethylbenzidine (TMB) substrate. Absorbance was measured at 450 nm, and measles-specific IgG activity was determined from the antigen-specific, control-corrected absorbance according to the manufacturer's quantitative procedure.

### Microbiome profiling

Fecal microbiota profiling by 16S rRNA gene sequencing was previously published.^30^ In brief, DNA was extracted from rectal swabs using the QIAamp Powerfecal DNA isolation kit (Qiagen INC, Hilden, Germany). Amplicons were generated for the 16S rRNA gene V3V4 variable region using primers 338F (5’ ACTCCTACGGGAGGCAGCAG) and 806R (5’ GGACTACHVGGGTWTCTAAT). Paired-end sequencing was performed on the Illumina MiSeq using a 600-cycle version 3 reagent kit. Sequence QA/QC and closed-reference sequence classification by SINA/Silva138^31^^,^^32^ using Last Common Ancestor assignment to the genus-level followed our previous publications.^33^^,^^34^ The median Goods coverage score was ≥99.8%, indicating excellent depth of sequence coverage.

### Ethics

This longitudinal cohort study was approved by the Human Research Ethics Committee at the University of the Witwatersrand (approval number: M171185) and the Colorado Multiple Institutions Review Board (COMIRB 17-0306). The study was conducted in accordance with applicable ethical requirements and was consistent with the ethical principles of the 2024 Declaration of Helsinki. Pregnant women with and without HIV admitted for delivery at Chris Hani Baragwanath Academic Hospital in Johannesburg, South Africa, were enrolled after providing written informed consent. Written parental informed consent was obtained for infant participation and sample collection. Informed consent procedures were designed after consultation with stakeholders for cultural sensitivity and included an open invitation to opt out of the study.

### Statistics

The primary outcome measures in this study were CMI and antibody responses to BCG, measles and tetanus in HEUs and HUUs and the primary predictors were Treg/Tici subsets and the composition of gut microbiome. Because sex, birth weight, maternal demographic characteristics, exclusive breastfeeding, maternal and infant diet and interval between the last dose of each vaccine and the immune response measurements might affect associations between outcome and predictor variables, these variables were included as covariates in analyses. The direct effects of these variables on Treg/Tici populations and gut microbiome parameters were analyzed and reported in our previous publications of this cohort.^19^^,^^30^ These analyses were novel and hypothesis-generating. Therefore, no a priori sample size calculation was performed.

The primary outcome measures were summarized using descriptive statistics and visualized in Prism version 10.1 for Mac (Graphpad). All inferential analyses of the associations between immune response and predictors were performed using multivariable models as described below. Analyses were performed using a complete-case approach because participant inclusion depended on the availability of biological specimens and corresponding assay results. We did not impute missing data because imputation would have required assumptions about the relationships among the variables and could have introduced additional uncertainty. Consequently, sample sizes varied across analyses. The number of participants included in each analysis is reported in the Results section and corresponding figure legends.

To analyze associations between immune responses to vaccines and their predictors, we used hurdle models for CMI to accommodate large proportions of zero responses and overdispersion of the non-zero responses. Moreover, the hurdle model allowed us to include potential confounders as covariates (e.g., sex, HEU status, breastfeeding, and time since the last dose of vaccine) as appropriate. The hurdle framework separately modeled the probability of a non-zero immune response (binary component via logistic regression), and the magnitude of the positive responses using a zero-truncated negative binomial model. p values for the coefficients were obtained using Wald Tests and were adjusted for multiple comparisons using the Benjamini-Hochberg false discovery rate (FDR) procedure. Assumptions were assessed by verifying that predictors exhibited approximately linear relationships with the logit of the binary outcome and with the log of the positive counts. Before testing the associations of CMI with the microbiome composition, genus-level 16S rRNA gene count data were transformed using the centered log-ratio (CLR) method to account for compositionality using the ALDEx2 package v1.38.0^35^ in R. The distribution of CLR-transformed variables was evaluated visually via histograms and Q–Q plots, which confirmed improved symmetry. However, normality was not required, as CLR variables were used only in hurdle models and Spearman correlations, neither of which assumes normality of predictors. Since BCG was administered at birth and previous studies of the neonatal gut microbiome did not find significant numbers of bacteria in infants delivered by C-section^36^^,^^37^ but revealed rapid colonization in the first few days of life with a mixture of maternal gut and vaginal microbiota,^38^, ^39^, ^40^, ^41^ CMI responses to BCG were correlated with maternal gut microbiota. Other immune responses were correlated with infant microbiota at the closest time point to vaccine administration. Gut microbiota Shannon diversity was calculated with the Explicet software package v3.3.22.^42^ Hurdle models assessed associations between CMI responses and demographic and HIV infection characteristics, Treg/Tici subsets with ≥0.001% frequency at any timepoint, CLR-transformed microbiome genus abundances, filtered by prevalence (≥5% of samples) and relative abundance (≥0.01% in at least one sample) and with Shannon diversity. Potential confounders, including sex, birth weight, maternal viral load and CD4 count at delivery in HEUs, exclusive breastfeeding, and the interval between the last vaccine dose and immune response measurement, were evaluated and included only when they materially affected the associations of interest.

Associations between measles IgG concentrations, which did not contain zero responses, and Treg/Tici frequencies, microbiome abundances, and diversity were evaluated using Spearman rank correlations. To remove confounding by HEU status, linear regression was used to generate HEU adjusted residuals for all variables. These residuals were then used in correlation analyses. Linear regression was used to generate HEU adjusted residuals and was not used for inference.

In a secondary analysis, microbiome enrichment calculated with the FGSEA package^43^ in R tested whether specific phyla were disproportionately associated with immune outcomes. For this analysis, genus-level associations from hurdle models and Spearman correlations were rank-ordered from most positive to most negative correlation coefficients. Phyla with <10 constituent genera were excluded from the analyses. An FDR p < 0.05 defined statistical significance. All analyses used Rv4.4.2.^44^

### Role of funders

The funders did not have any role in study design, data collection, data analysis, interpretation, or writing of report.

## Results

### Characteristics of the study population

The study enrolled 240 mother-infant pairs from Soweto between June and December 2017, including 123 mothers with HIV and 117 without HIV (Table 1). Mothers with HIV received ART, consisting mostly of efavirenz, tenofovir disoproxil fumarate, and lamivudine during pregnancy, had a median of 347 CD4+ T cells/μl of blood and <50 HIV RNA copies/ml of plasma at delivery.

**Table 1:** Demographic characteristics of the study population.

Variable^a^	HEU N = 123	HUU N = 117	p-value^b^
Maternal			
Age (years)	30 (26, 35)	25 (22, 30)	<0.001
Previous pregnancies	2.00 (1.00, 3.00)	1.00 (0.00, 2.00)	<0.001
Missing	2	5	
Smoking during pregnancy	9 (7.3%)	4 (3.4%)	0.3
Alcohol during pregnancy	11 (8.9%)	8 (6.8%)	0.7
CD4 count	347 (227, 503)	NA	
Missing	7		
CD4 Count≥200	94 (81%)	NA	
Missing	7		
Log HIV RNA Copies/ml^c^	1.00 (0.00, 2.06)	NA	
Log HIV RNA Copies/ml≤1.3	70 (62%)	NA	
Missing	10		
Infant			
Sex			0.5
Male	58 (47%)	61 (52%)	
Female	65 (53%)	56 (48%)	
Vaginal delivery	116 (94%)	116 (99%)	0.084
Gestational age	39.00 (38.00, 40.00)	40.00 (38.00, 40.00)	0.056
Missing	4	1	
Gestational age≥37 weeks	117 (98%)	113 (97%)	>0.9
Missing	4	1	
Birth weight (g)	3090 (2,825, 3365)	3275 (2,965, 3560)	0.002
Birth weight≤2500 g	8 (6.5%)	7 (6.0%)	>0.9

Abbreviations: ART = antiretroviral treatment; BMI = Body mass index; HIV = Human immunodeficiency virus; HEU = HIV-exposed uninfected; HUU = HIV-unexposed uninfected.

a Median (Q1, Q3); n (%).

b Wilcoxon rank sum test; Pearson's Chi-squared test.

c Target not detected was assigned a numeric value of 0 and < 20 a value of 10 copies/ml.

At birth, HEUs and HUUs had similar gestational ages with an average of 39 weeks. The sex distribution was also similar (Table 1). HEUs had significantly lower birth weight with a mean of 3070 g compared with 3250 g in HUUs, but the difference was not biologically relevant. Seven HEUs and one HUU were delivered by emergency C-section for obstetrical indications identified after initiation of labor.

Over 80% of the HEUs and HUUs were breastfed during the first 6 months of life, including exclusive breastfeeding or mixed with other products (Table 2). HEUs received cotrimoxazole up to two months after cessation of breastfeeding per contemporaneous WHO recommendations.

**Table 2:** Feeding pattern and cotrimoxazole use in HEUs and HUUs.

	6 Weeks HEU (N = 98)	6 Weeks HUU (N = 88)	28 Weeks HEU (N = 86)	28 Weeks HUU (N = 80)	62 Weeks HEU (N = 74)	62 Weeks HUU (N = 74)
Feeding method						
Exclusive breast feeding	83 (84.7%)	72 (81.8%)	52 (60.5%)	40 (50.0%)	0 (0%)	0 (0%)
Exclusive formula feeding	13 (13.3%)	4 (4.5%)	19 (22.1%)	9 (11.3%)	0 (0%)	0 (0%)
Mixed^a^	2 (2.0%)	12 (13.6%)	15 (17.4%)	31 (38.8%)	74 (100%)	74 (100%)
Cotrimoxazole						
No	93 (94.9%)	88 (100%)	8 (9.3%)	79 (98.8%)	10 (13.5%)	74 (100%)
Yes	5 (5.1%)	0 (0%)	77 (89.5%)	0 (0%)	64 (86.5%)	0 (0%)
Missing	0 (0%)	0 (0%)	1 (1.2%)	1 (1.3%)	0 (0%)	0 (0%)

a Any combination of breastmilk, formula, and/or solids.

Vaccines were administered as per the South African Expanded Programme of Immunization, which recommends BCG at birth, tetanus at 6, 10 and 14 weeks of life and measles at 26 and 52 weeks of life.^45^ We analyzed responses to vaccines after the last dose in participants with complete immunization regimens. There were no significant differences in the time of administration of the last dose of vaccine between HEU and HUU (Table 3).

**Table 3:** Weeks of age at vaccine administration.

Variable^a^	HEU N = 123	HUU N = 117	p-value^b^
BCG	0.00 (0.00, 0.00)	0.00 (0.00, 0.00)	0.2
Unknown	1	1	
First tetanus	6.14 (6.00, 6.71)	6.00 (6.00, 6.50)	0.3
Unknown	36	33	
Second tetanus	10.57 (10.00, 11.14)	10.29 (10.00, 10.86)	0.069
Unknown	40	42	
Third tetanus	14.71 (14.14, 15.86)	14.71 (14.00, 15.86)	0.5
Unknown	40	40	
First measles	26.71 (26.29, 27.86)	26.29 (26.00, 26.93)	<0.001
Unknown	52	41	
Second measles	52.43 (52.14, 52.86)	52.57 (52.14, 53.00)	0.2
Unknown	50	44	

a Median (Q1, Q3).

b Wilcoxon rank sum test.

### CMI and humoral responses to BCG, tetanus toxoid, and measles vaccines administered to HEUs and HUUs in the first year of life

CMI responses to BCG were measured by IFNγ and IL-2 dual-color FluoroSpot in peripheral blood mononuclear cells (PBMCs) obtained at 6 weeks of life using an *M. tuberculosis* antigen preparation (Mtb). CMI testing was performed in 23 HEU and 21 HUU BCG recipients who did not have household exposure to tuberculosis before 6 weeks of age and had available PBMCs. Demographic characteristics of the HEUs and HUUs who contributed data to the analysis of CMI responses to BCG (Table S1) did not appreciably differ from those of the entire cohort. At 6 weeks of life, only 17% of HEU and 43% of HUU infants had measurable Mtb-specific IFNγ responses (Fig. 1A). Using the hurdle model, as appropriate for the large number of nonresponders, we performed a multivariable analysis of the BCG CMI adjusted for HEU status and other variables previously shown to affect measures of immune responses to vaccines, including sex and breastfeeding. The adjustment was performed by including these variables as covariates in the model. The results showed a non-significant negative effect of HEU status on the frequency of IFNγ responses to Mtb (hurdle model binary component, p = 0.07). The magnitude of IFNγ responses was also lower in HEU (mean = 12 SFC/10^6^ PBMC) than HUU infants (mean = 55 SFC/10^6^ PBMC) without reaching statistical significance (Fig. 1B). IL-2 responses to Mtb did not appreciably differ between HEUs and HUUs (Fig. 1B). Sex did not affect the CMI responses to BCG, but exclusive breastfeeding was associated with lower IFNγ responses (effect estimate = −1.7; hurdle model magnitude component, FDR p = 0.007). In HEU infants, we also evaluated the potential effect of the maternal HIV infection characteristics on the responses to the vaccine. Correlation analyses of Mtb-specific IFNγ and IL-2 responses at 6 weeks of life with maternal CD4 cell numbers and HIV RNA copies/ml of plasma at delivery did not show significant associations (Table S2).

**Fig. 1:**
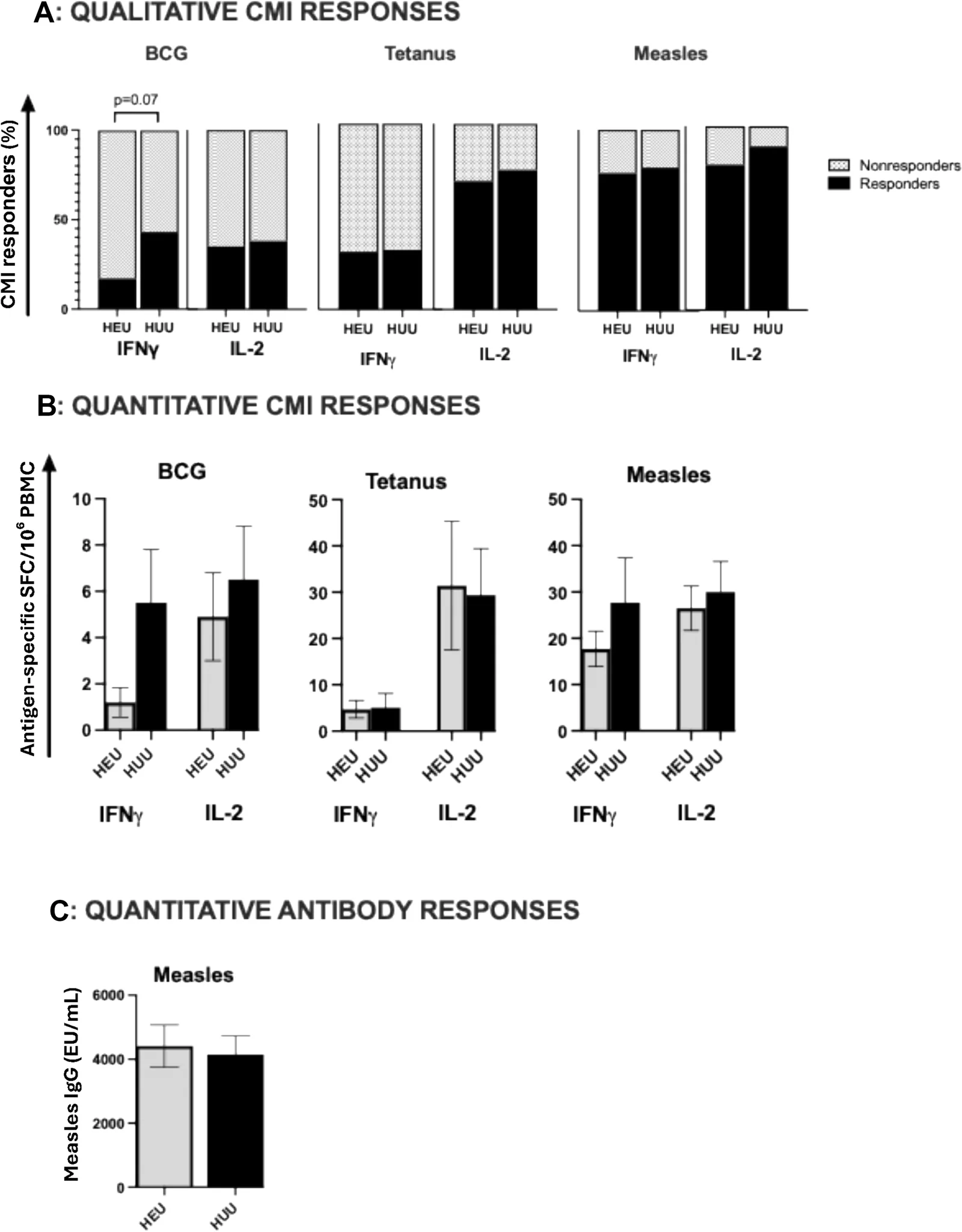
**Immune responses to childhood vaccines in HEU compared to HUU infants.** Data were derived from 23 HEUs and 21 HUUs with data on CMI responses to BCG at 6 weeks of life; 29 and 28 with data on CMI responses to measles and tetanus at 62 weeks of life; 66 and 68 with measles antibody concentrations measured at 62 weeks of life. **Panel A** shows qualitative CMI responses; **Panel B** shows quantitative CMI responses; **Panel C** shows quantitative antibody responses. Statistical comparisons used the Hurdle model magnitude and binary components adjusted for sex, breastfeeding, and time since vaccination as appropriate. p values < 0.1 are shown on the graphs.

CMI responses to tetanus and measles vaccines were measured at 62 weeks of life. We tested PBMC from 29 HEU and 28 HUU infants with documented receipt of three doses of tetanus and two doses of measles vaccines. The time elapsed between the last dose of vaccine and the day of testing had a median (min; max) of 80 days (40; 90) for tetanus and 43 days (2; 52) for measles vaccines. Demographic characteristics of the participants who contributed data to this analysis did not appreciably differ from the characteristics of the entire cohort (Table S3).

The hurdle multivariable model for tetanus CMI responses, controlling for the effect of HEU status, sex, time from the last dose of vaccine, and exclusive breastfeeding at the last dose administration, showed the following: (1) similar proportions of responders and magnitude of IFNγ and IL-2 responses in HEUs and HUUs (Fig. 1A); (2) higher IFNγ and IL-2 responses to tetanus toxoid in males than females (effect sizes = 1.3 and 0.8; hurdle model magnitude component, p = 0.0007 and 0.007, respectively); (3) lower IL-2 responses in association with longer interval since the last dose of vaccine (effect size = −0.03, hurdle model magnitude component; p = 0.10); and (4) lower IFNγ responses in exclusively breastfed infants at the last dose of vaccine (effect size = −0.88; hurdle model magnitude component, FDR p = 0.008).

The hurdle model for measles CMI controlled for HEU status, time since the last dose of vaccine and sex. Exclusive breastfeeding was not included in the model because none of the participants was exclusively breastfed when the last dose of measles vaccine was administered. The analysis showed the following: (1) no significant differences in the proportion of responders or the magnitude of IFNγ and IL-2 responses between HEUs and HUUs (Fig. 1A); (2) lower responses in association with longer time since the last dose of vaccine both for IL-2 (effect size = −0.04; hurdle model magnitude component, FDR p = 0.005) and IFNγ (effect size = −0.04; hurdle model magnitude component, FDR p = 0.03); and (3) no effect of sex.

Antibody responses to measles measured at 62 weeks of life in 66 HEUs and 68 HUUs with demographic characteristics similar to those of the entire cohort (Table S4) showed similar antibody concentrations in the two groups after adjustment for sex and time since the last dose of vaccine (multivariable regression model, FDR p = 0.99; Fig. 1). Sex did not affect the antibody concentrations. Time since the last dose of vaccine was negatively associated with the antibody concentrations (effect size = −0.79; multivariable regression model, FDR p = 0.0002).

### Treg and Tici frequencies are significantly associated with the magnitude of cell-mediated and humoral immune responses to vaccines in HEUs and HUUs

We analyzed the relationship of Mtb-specific IFNγ and IL-2 FluoroSpot results at 6 weeks of life, tetanus toxoid- and measles-specific CMI responses and measles antibody responses at 62 weeks of life with the frequencies of Treg and Tici subsets measured at the study visit closest to the last dose of each vaccine (birth for BCG, week 28 for tetanus and week 62 for measles). Treg and Tici frequencies were measured in two panels, including CD3, CD4, CD25, CTLA-4, FOXP3, GranzB, IL-10, LAG-3, and TGFβ markers in panel A; and CD3, CD4, CD39, FOXP3, GITR, IL-35, PD-1, TIGIT, and TNFr2 in panel B (gating strategy shown in Figure S1). After exclusion of Ticis or Tregs with <0.001% events in both HEUs and HUUs, 25 T cell phenotypic subsets were included in the statistical analysis.

BCG was administered in a single dose at birth. Hence, we investigated the effect of Tregs and Ticis measured at birth on Mtb-specific CMI measured at 6 weeks of life. The multivariable hurdle model using data from 23 HEUs and 16 HUUs with paired FluoroSpot and flow cytometry results revealed a significant negative effect of the frequency of CD4+CD39+ Treg (hurdle model magnitude component, FDR p = 0.04) and a positive effect of CD4+IL-35+ Treg on Mtb-specific IFNγ responses (hurdle model magnitude component, FDR p < 0.0001; Fig. 2A). The frequency of CD4+CTLA4+, CD8+CD39+, CD8+PD1 and CD8+FOXP3+ T cell subsets also had negative effects on the IFNγ responses to BCG without reaching statistical significance after FDR correction (hurdle model magnitude component, FDR p = 0.06–0.10; Fig. 2A). There were no significant effects of Tregs or Ticis on Mtb-specific IL-2 responses. HEU status did not have a significant effect in the multivariable model suggesting that Tregs and Ticis may have at least partially accounted for the differences in BCG-IFNγ responses between HEUs and HUUs.

**Fig. 2:**
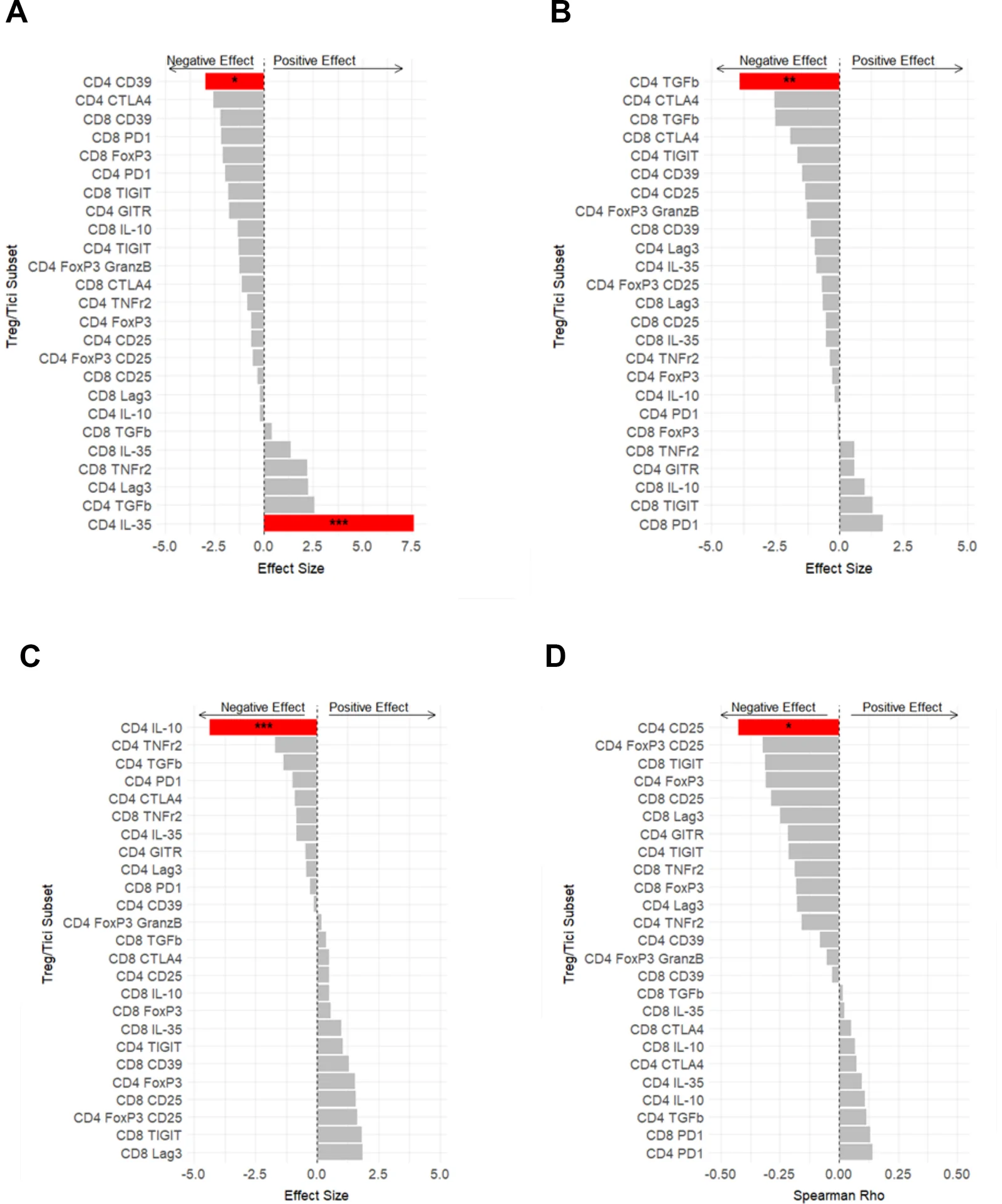
**Effect of Tregs and Ticis on HEUs and HUUs CMI and antibody responses to vaccines.** Graphs show Forest plots of the effect of Treg/Tici on BCG IFNγ CMI responses in 23 HEUs and 16 HUUs (**Panel A**) Tetanus IL-2 CMI responses in 23 HEUs and 16 HUUs (**Panel B**); Measles IL-2 CMI in 17 HEUs and 11 HUUs (**Panel C**); and Measles antibody responses in 31 HEUs and 28 HUUs (**Panel D**). The outcome measure is indicated in the graph title and the T cell subsets are listed on the y axes. The x axes show effect sizes. Red bars highlight significant effects with FDR p values < 0.05 and include the magnitude of the p values as specified by asterisks: ∗p < 0.05; ∗∗p < 0.01; ∗∗∗p < 0.001. p values for Panels A–C were obtained using Wald tests of hurdle-model coefficients (magnitude component); Panel D used Spearman rank correlation. p values were adjusted for multiple comparisons using the Benjamini-Hochberg FDR procedure. Analyses controlled for HEU/HUU status of the participants.

We next studied the relationship of tetanus toxoid-specific CMI responses with Tregs and Ticis measured at 28 weeks of life, the closest timepoint to the 3rd and last dose of the tetanus vaccine, in 23 HEUs and 16 HUUs. The analysis showed a significant negative effect of the frequency of CD4+TGFβ+ Tregs on tetanus-toxoid IL-2 responses (hurdle model magnitude component, FDR p = 0.003; Fig. 2B), but not on IFNγ responses. CD4+CTLA4+ and CD8+TGFβ+ Tregs had marginal negative effects on IL-2 responses to tetanus toxoid (hurdle model magnitude component, FDR p = 0.10; Fig. 2B). The HEU status of the participants did not have an effect.

The analysis of measles-specific CMI responses with Tregs and Ticis measured in 17 HEUs and 11 HUUs with paired FluoroSpot and flow cytometry results at 62 weeks of life, which was the closest time point to the administration of the 2nd and last dose of measles vaccine, showed a significant negative effect of CD4+IL10+% on IL-2 responses (hurdle model magnitude component, FDR p = 0.0004; Fig. 2C). There was no effect of Treg or Tici frequencies on IFNγ responses. The HEU status did not show an appreciable effect in the model.

The effect of Treg and Tici frequencies at the time of the last dose of measles vaccine on measles antibody responses was examined in 31 HEUs and 28 HUUs with paired data. The analysis showed a significant negative association of CD4+CD25+ Tregs with the antibody concentrations (Spearman rank correlation, FDR p = 0.04: Fig. 2D) after controlling for HEU status. The frequencies of CD4+CD25+FOXP3+ and CD8+TIGIT+T cell subsets had non-significant negative effects on antibody concentrations (Spearman rank correlation, FDR p = 0.11; Fig. 2D). The HEU status did not have appreciable effects.

Individual Spearman correlation analyses in HEUs and HUUs did not provide any information that was not already gleaned from the adjusted hurdle or linear regression models. The only significant association in these analyses was a positive correlation between tetanus IL-2 and CD8+LAG3+Tici in HEU with Rho = 0.68 and FDR p = 0.097.

### The composition of the gut microbiome is significantly associated with the immunogenicity of vaccines in HEUs and HUUs

We investigated the relationship of antibody and/or CMI responses to BCG, tetanus and measles vaccines with 225 bacterial genera identified by 16S rRNA gene sequencing in the maternal gut at delivery or in the infant gut at the closest study visit to the last dose of each vaccine as described for Tregs and Ticis. We performed analyses at genus-level complemented by enrichment analyses of the phyla with ≥10 genera in the gut microbiome of our participants, represented by Actinobacteria, Bacteroidetes, Firmicutes and Proteobacteria. We also investigated the effect of alpha diversity (Shannon H index) on responses to vaccines. All analyses controlled for HEU status.

We analyzed the relationship of the maternal gut microbiome at delivery with CMI responses to BCG in 23 HEUs and 20 HUUs with paired CMI and microbiome results. The genus-level analysis revealed significant effects of 22 bacterial taxa on IL-2 responses, including 11 Firmicutes and 4 Actinobacteria with negative effects, and 4 Proteobacteria, 2 Bacteroidetes and 1 Actinobacterium with positive effects (Fig. 3A). There were no individual genera significantly associated with IFNγ responses to BCG. The relative abundance of the Firmicute *Murdochiella sp*. had a significant differential effect on the magnitude of IL-2 responses to BCG in HEUs compared with HUUs (effect size = 1.2, hurdle model magnitude component, FDR p < 0.0001; Fig. 3C). The analysis of the gut microbiome composition at 28 weeks of life, closest to the last dose of tetanus vaccine, on 27 HEUs and 27 HUUs with paired data did not identify bacterial genera significantly associated with tetanus CMI. The genus-level analysis of the relationship with responses to the measles vaccine in 26 HEUs and 27 HUUs with paired microbiome and FluoroSpot results at 62 weeks of life (closest study visit to the last dose of measles vaccine) revealed 5 bacterial taxa with negative effects on IL-2 responses, including 2 Firmicutes, 2 Proteobacteria and 1 Tenericute (hurdle model magnitude component, FDR p ≤ 0.046; Fig. 3B). There were no associations with the IFNγ or antibody responses to the measles vaccine. We did not find differential effects of the microbial genera on HEU compared with HUU immune responses to tetanus or measles vaccines.

**Fig. 3:**
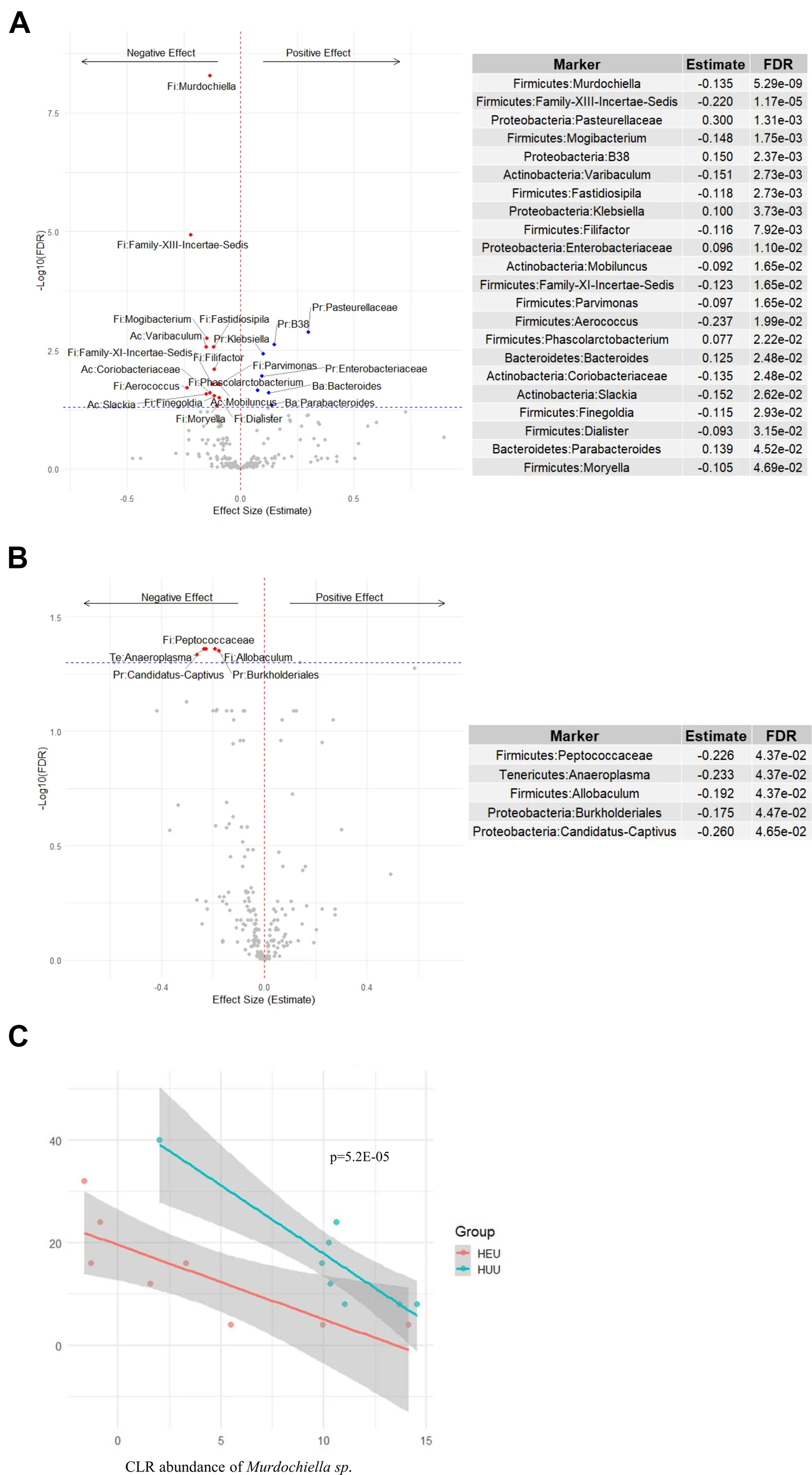
**Effect of gut microbial genera on infant responses to vaccines.** The volcano plots show results from the magnitude component of the hurdle models, with genera having significant positive effects at FDR p < 0.05 in blue and significant negative effects at FDR p < 0.05 in red. Gray indicates effects with FDR p ≥ 0.05. The tables show genera with significant effects corresponding to each plot. Analyses controlled for HEU/HUU status of the participants. **Panel A** shows associations of bacterial genera with IL-2 responses to BCG in 23 HEUs and 20 HUUs. **Panel B** shows associations of bacterial genera with IL-2 responses to measles in 27 HEUs and 27 HUUs. **Panel C** shows the correlation of Mtb-specific IL-2 responses of 8 HEUs and 8 HUUs with positive ELISPOT results with the centered log-ratio (CLR) transformed abundance of *Murdochiella sp.* The graph shows individual participant datapoints, the linear regression line and 95% confidence intervals, and the FDR p-value. p values were obtained using Wald tests.

The enrichment analysis showed significant negative effects of Actinobacteria (FGSEA, FDR p = 0.002) and Firmicutes (FGSEA, FDR p = 0.02) on IL-2 responses to BCG; significant positive effects of Proteobacteria both on IFNγ and IL-2 responses (FGSEA, FDR p of 0.01 and < 0.0001, respectively); and a marginal positive effect of Bacteroidetes on IL-2 responses (FGSEA, FDR p = 0.07; Fig. 4A and B and Figure S2). The microbiome enrichment analysis at 28 weeks of life (closest study visit to the 3rd dose of tetanus vaccine) showed a significant negative effect of Actinobacteria on IL-2 responses to tetanus (FGSEA, FDR p = 0.04) and a marginal effect on IFNγ responses (FGSEA, FDR p = 0.10; Fig. 4C and D and Figure S2). Bacteroidetes had a marginal positive effect on IFNγ (FGSEA, FDR p = 0.10; Fig. 4D and Figure S2). The enrichment analysis of measles CMI and the gut microbiome showed a marginal positive effect of Proteobacteria on IL-2 responses (FGSEA, FDR p = 0.10; Fig. 4E and Figure S2) and no effect on IFNγ. With respect to measles antibody responses, the gut microbiome enrichment analysis in 59 HEUs and 69 HUUs with paired antibody and microbiome data revealed a negative effect of Actinobacteria (FGSEA, FDR p = 0.008) and a positive effect of Proteobacteria (FGSEA, FDR p = 0.02; Fig. 4F and Figure S2).

**Fig. 4:**
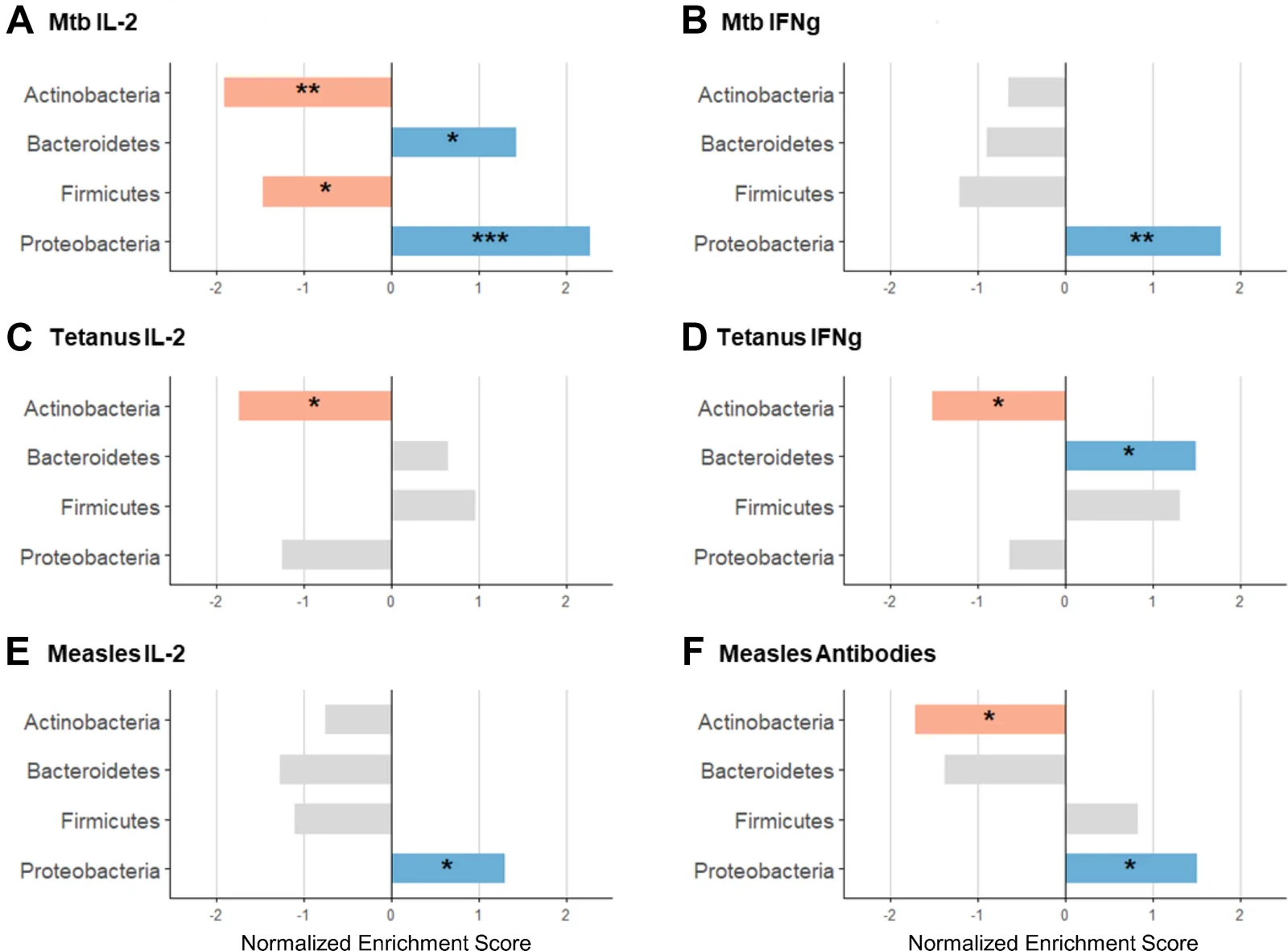
**The effect of gut microbial phyla enrichment on infant responses to vaccines.** The graphs show the normalized enrichment score (NES) for the outcome measure indicated in the panel title. Enrichment analyses included phyla with ≥10 genera in the study participants. Blue bars indicate positive NES, red bars indicate negative NES. Asterisks indicate the FDR p values: ∗p < 0.05; ∗∗p < 0.01; ∗∗∗p < 0.001. All analyses controlled for the HEU/HUU status of the participants. **Panels A** (IL-2) **and B** (IFNγ) used data on responses to BCG measured at 6 weeks of life in 23 HEUs and 20 HUUs. **Panels C** (IL-2) **and D** (IFNγ) used data on responses to tetanus vaccine measured at 62 weeks of life in 27 HEUs and 27 HUUs. **Panel E** (Measles IL-2) used data from 27 HEUs and 27 HUUs. **Panel F** (Measles Antibodies) used data from 59 HEUs and 69 HUUs.

Higher Shannon diversity was significantly associated with lower magnitude of IL-2 responses to BCG and lower proportions of IFNγ BCG responders (hurdle model, p = 0.002 (magnitude component) and 0.03 (binary component), respectively; Fig. 5A and B). The association of the Shannon diversity index with IFNγ response to BCG was more prominent in HEUs than HUUs (hurdle model binary component, p = 0.047; Fig. 5C). Diversity was not associated with the CMI responses to tetanus vaccine. High Shannon diversity indices were associated with lower IFNγ responses to the measles vaccine (hurdle model magnitude component, p = 0.004; Fig. 5D) without a differential effect by HEU status. We did not find significant associations of measles antibody concentrations with the richness and diversity of the microbiota (Spearman rank correlation, rho = −0.15, FDR p = 0.17).

**Fig. 5:**
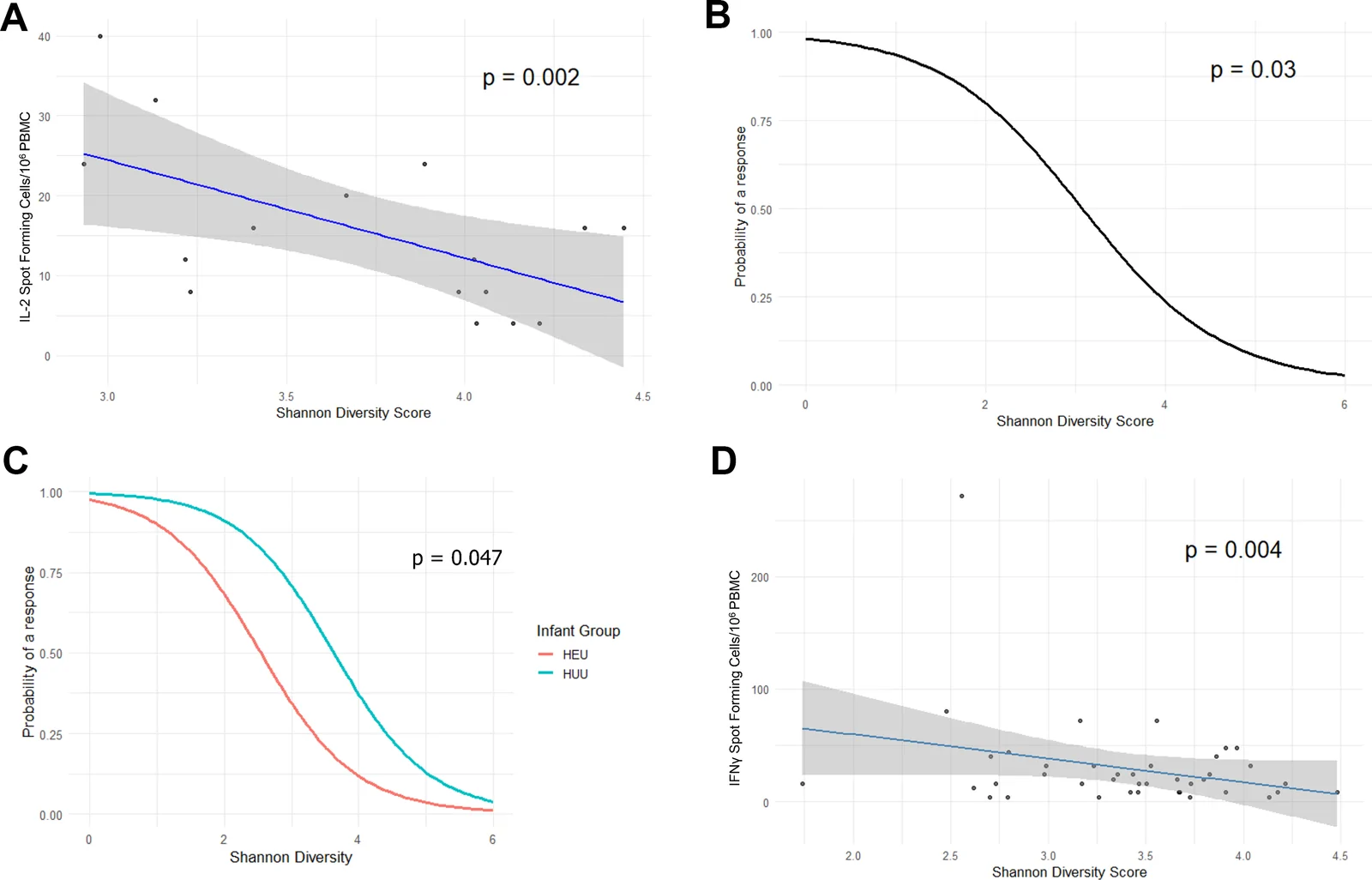
**The effect of the gut microbiome diversity on infant responses to vaccines. Panel A** shows correlations between quantitative IL-2 responses to the BCG vaccines and Shannon diversity score shown on the x axis in 23 HEUs and 20 HUUs. p-value calculated by hurdle model. **Panel B** shows the relationship between qualitative IFNg responses to BCG vaccine and Shannon diversity. For each unit increase in Shannon diversity, the log odds of having a response decreases by 1.43. p-value calculated by hurdle model. **Panel C** shows the differential effect of Shannon diversity on IFNγ responses to BCG in HEUs and HUUs. **Panel D** shows the relationship between quantitative IFNγ responses to measles vaccine and Shannon diversity in 27 HEUs and 27 HUUs. Panels A and D used the magnitude component and Panels B and C used the binary component of the hurdle model; p values were obtained using Wald tests.

## Discussion

Responses to vaccines have been used as a surrogate marker of immune system functionality. To determine if decreased CMI responses to vaccines might provide insight into the immunological defects that account for the increased susceptibility to severe infections in HEUs compared with HUUs, we measured responses to three vaccines administered at different timepoints and in different number of doses during the first year of life. We showed that CMI responses to BCG, which is administered in a single dose at birth, were lower in HEUs compared with HUUs. In contrast, CMI responses to tetanus and measles vaccines, administered in 3 and 2 doses, respectively, with last doses at ≥6 months of age did not differ between HEUs and HUUs. Our results agree with other studies that showed significantly lower responses to vaccines in HEUs than HUUs only in the first few months of life.^9^^,^^46^ Collectively, these findings suggest that CMI is significantly impaired in HEUs in the first few months of life after which differences with HUUs are attenuated. Notably, the increased infectious morbidity in HEUs also peaks in the first 3–6 months of life.^47^, ^48^, ^49^, ^50^, ^51^ An alternative explanation for the decreased CMI after BCG, but not after tetanus or measles vaccines is that we measured the primary response to BCG and recall responses to tetanus toxoid and measles. It is well known that influenza, RSV and other infections that rely on CMI for pathogen eradication are most severe after primary exposures. Collectively, this information suggests that future studies of immunologic dysfunctions of HEUs could focus mainly on primary responses in the first 6 months of life.

We hypothesized that the composition of the gut microbiome at the time of vaccination affects the immunogenicity of vaccines and may contribute to the difference in the immune responses of HEUs and HUUs. We determined that high relative abundances of Actinobacteria and Firmicutes at the time of the last dose of vaccines were generally associated with decreased CMI and antibody responses measured weeks to months after immunization both in HEUs and HUUs. In contrast, high relative abundance of Bacteroidetes and Proteobacteria generally had positive effects on the magnitude of CMI and humoral responses to the vaccines. Although we studied the immunogenicity of both live attenuated (BCG and measles) and inactivated subunit vaccines (tetanus toxoid) we found a remarkable consistency of the effect of Actinobacteria, Bacteroidetes, and Proteobacteria on vaccine immunogenicity. The genus-level analysis generally confirmed the enrichment findings. Moreover, high diversity of gut microbiota was associated with decreased CMI responses to both BCG and measles vaccines. Previous studies have yielded heterogeneous results on the effect of the gut microbiome on immunogenicity of vaccines. Studies that analyzed the gut microbiome and vaccine responses simultaneously failed to identify significant associations.^52^, ^53^, ^54^ In contrast, investigations like ours that analyzed the gut microbiome at the time of vaccination found significant associations.^54^, ^55^, ^56^ Since the infant gut microbiome continuously changes in the first year of life, the gut microbiome composition is likely to differ several months after vaccination compared to the composition at the time of vaccination explaining the discrepant results.^57^ Both our study and previous investigations of the gut microbiome close to the time of vaccination, identified positive effects of the abundance of Bacteroidetes on the immunogenicity of vaccines.^55^^,^^56^ In contrast, we found a negative effect of Actinobacteria, while other investigators found positive effects of the Actinobacteria *B. spp.* or *Bifidobacterium longum.* In our study, *B. spp.* did not show significant associations with the immunogenicity of vaccines. Differences in the study designs may explain the discrepancy of the results, including the geographic location of the participants, which was previously shown to have a profound effect on the gut microbiome,^58^ the inclusion of HEUs in our study, but not in other studies, and the fact that we analyzed the gut microbiome at the time of the last dose of vaccine, whereas other studies analyzed the gut microbiome in early infancy when the first and second doses of childhood vaccines are typically administered.^54^, ^55^, ^56^ The mechanisms underlying the effect of the gut microbiome on the immunogenicity of vaccines are probably diverse and need additional studies to fully understand them. They may involve specific bacterial metabolites and antigens crossing into the circulation and contributing to immune cell activation or regulation in a way that modulates responses to vaccines.^24^^,^^59^^,^^60^ However, regardless of the mechanisms of action, our findings suggest that there may be an opportunity to improve responses to vaccines in infants through interventions targeting the gut microbiome.^61^

We identified gut microbiome characteristics differentially associated with HEU and HUU responses to vaccines. These included the relationship of *Murdochiella sp.* abundance and of Shannon diversity with responses to BCG. BCG was the only vaccine in this study that generated lower responses in HEUs compared with HUUs, which may explain why the differential effect of the microbiome composition was detected for BCG but not for other vaccines. There are very few studies on the effect of *Murdochiella* on immune responses. When present in the penile or vaginal microbiome together with other anaerobic bacteria, including *Prevotella, Dialister, Peptoniphilus,* and *Negativicoccus,* it was positively associated with inflammation and increased HIV acquisition.^62^, ^63^, ^64^ In our study, the abundance of *Murdochiella* in the maternal gut microbiome was associated with lower Mtb-specific IL-2 responses in all infants, with a greater effect in HEUs. The Shannon diversity index was also negatively associated with Mtb-specific IFNγ qualitative responses overall but had a more prominent effect in HEUs than HUUs. These associations may have contributed to the lower immune responses in HEUs than HUUs.

In our previous studies, we found that HEUs and HUUs differed with respect to the proportions of Tregs and Ticis in cord and peripheral blood during the first year of life^19^ and postulated that these cell subsets may contribute to the decreased immune function of HEUs. Both Tregs and Ticis have been demonstrated to hamper immune responses to tumors, infections, and vaccines in other studies.^65^, ^66^, ^67^, ^68^ We found that high frequencies of CD4+CD39+, CD4+IL10+, CD4+TGFβ+, and CD4+CD25+ Treg subsets were significantly associated with decreased CMI and/or antibody responses to BCG, tetanus, and/or measles vaccines. High frequencies of CD4+CTLA4+ Treg had marginal negative effects on IFNγ responses to BCG and tetanus vaccine, which is notable because it was the only Treg subset to impact responses to more than one vaccine. Other T cell subsets with marginal negative effects on responses to vaccines were CD4+CD25+FOXP3+ and CD8+FOXP3+ Treg and CD8+PD1+ and CD8+TIGIT+Tici. Unexpectedly, CD4+IL35+ Treg frequencies were positively associated with IFNγ responses to BCG. Importantly, although the Tregs and Ticis did not show a differential effect on HEU versus HUU responses to vaccines, the inclusion of Tregs and Ticis in the model eliminated the difference in BCG-IFNγ responses between HEUs and HUUs, suggesting that Tregs and/or Ticis may have contributed to this difference.

Since we previously showed an association between Tregs and gut microbiota that differentiated HEUs and HUUs,^19^ a goal of the current study was to determine if Tregs may mediate the effect of the microbiome on the immunogenicity of vaccines in HEUs. However, the Treg subsets and the bacterial taxa whose increased frequencies or abundances were associated with lower immunogenicity of vaccines were not correlated among themselves in our previous study.^19^ This finding suggests that the Treg and gut microbiome effects on vaccine responses are likely to be independent.

Other factors that correlated with immune responses to vaccines included male sex, which was associated with higher responses against the tetanus vaccine. Sex has been shown to affect responses to several vaccines, but not all.^69^ Moreover, the findings have not been consistent across studies.^69^ Most studies reported higher antibody responses to tetanus vaccines in males than females, which is in agreement with our observation. The mechanism underlying the effect of sexual dimorphism on immune responses to vaccines is multifactorial, including genetic, hormonal, nutritional and other environmental factors.^70^ These factors need to be further explored to separate biological factors that may not be susceptible to interventions from environmental factors, such as nutrition, that could potentially be modulated.

Longer time since vaccination was associated with lower responses to measles and tetanus vaccines in our study participants. This finding was not unexpected considering the kinetics of recall antibody and CMI responses to vaccines, which peak in the first 2–4 weeks post-immunization, decay relatively rapidly in the subsequent 6–12 months and much slower thereafter.^71^

We did not find evidence that the maternal HIV infection characteristics at delivery, such as CD4+ T cell numbers and HIV plasma viral load, made appreciable contributions to CMI responses to vaccines in HEUs. We undertook this analysis because recent studies showed associations of infant growth and neurodevelopment with maternal HIV infection characteristics.^72^^,^^73^ However, this did not seem to be the case with immunogenicity of vaccines.

Exclusive breastfeeding was associated with lower CMI responses to BCG and tetanus vaccines in our study. Previous investigations found increased antibody responses to vaccines in breastfed compared with formula fed children with the exception of oral live vaccines whose replication may be impaired by pathogen-specific maternal IgA in the breastmilk.^69^ In contrast, there is little information on the effect of breastfeeding on CMI responses to vaccines. A study in mice found that maternal anti-SARS CoV-2 antibodies crossed the intestinal barrier and effected rapid clearance of the infant SARS CoV-2 vaccine antigens in the pups and inhibited their T cell responses.^74^ However, the intestinal mucosa in mice remains permeable to macromolecules such as maternal IgG for many weeks post-delivery, whereas in humans the permeabilities ceases in the first few days of life.^75^ Notably, human breastmilk contains many cytokines, including the regulatory cytokines TGF-β and IL-10, which are essential in tolerizing the neonate to food allergens but may also affect their systemic immune responses^76^ providing a plausible mechanism of inhibition of T cell responses to vaccines administered during breastfeeding. Additional studies are needed to fully elucidate the mechanism/s whereby breastfeeding alters CMI responses to vaccines.

Our study has limitations. The number of participants with PBMCs available both for FluoroSpot and flow cytometry assays was limited by the amount of blood that we were able to obtain from young infants, reducing the sensitivity of our analyses. In addition, because analyses were restricted to participants with complete assay data, the potential for selection bias cannot be excluded. The study enrolled participants only from South Africa and, therefore, our results may not be applicable to other geographic regions. Moreover, all HEUs received cotrimoxazole for up to 2 months after the cessation of breastfeeding, which in some cases extended beyond the first year of life. Cotrimoxazole is likely to have altered the gut microbiome of HEUs.^30^^,^^77^ Parasitologic exams were not performed so we cannot completely rule out asymptomatic parasitic infestations altering the gut microbiome.

In conclusion, the immunogenicity of vaccines was significantly correlated with the composition of the gut microbiome and with the relative abundance of Tregs and Ticis and may have contributed to the differential immune responses of HEUs and HUUs. The consistent effect of the gut microbiome composition at the time of vaccination on the immunogenicity of multiple vaccines suggests that dietary interventions may be attempted to enhance infant immune responses. Notably, since there is a strong correlation between maternal and infant gut microbiota,^30^ these interventions could be applied to the mother or infant.

## Contributors

MJJ: assay performance, data analysis and interpretation, manuscript writing, preparation of figures, manuscript review and approval. DNF: data analysis and interpretation, manuscript writing, manuscript review and approval. ATS: assay performance, manuscript review and approval. MCN: participant enrollment, demographic and clinical data collection, accessed and verified underlying data, manuscript review and approval. SAM: participant enrollment, accessed and verified underlying data, manuscript review and approval. AW: conceptualization, data analysis and interpretation, manuscript writing, manuscript review and approval. All authors read and approved the final version of the manuscript.

## Data sharing statement

The data and code that support the findings of this study are openly available in Zenodo at http://doi.org/10.5281/zenodo.16801770. All 16S rRNA gene sequences and corresponding metadata were deposited in the NCBI Sequence Read Archive under BioProject accession number PRJNA816484.

## Declaration of interests

AW reports grants from the NIH; consulting fees from Curevo, Seqirus, GlaxoSmithKline, and Pfizer; honoraria from Seqirus; and participation on a Curevo Data Safety Monitoring Board or Advisory Board. MCN reports a grant from Sanofi paid to her institution; consulting fees and honoraria from Pfizer paid to her institution; payments for expert testimony from Sanofi; support for attending meetings and/or travel from Sanofi and Pfizer; and a leadership or fiduciary role on the Resvinet board. SAM reports grants from the Gates Foundation, Pfizer, Minervax, and Merck paid to his institution; honoraria for lectures from Sanofi and Pfizer; and participation on a Bavarian Nordic advisory board for an mpox vaccine in pregnant women. MJJ, DNF, and ATS declare no competing interests.
