# Supplementary material for: Association of gut microbiota and regulatory T cells with vaccine immunogenicity in HIV-exposed uninfected and HIV-unexposed infants: a longitudinal cohort study
Source: eBioMedicine. 2026 Oct 1;132:106500. doi: 10.1016/j.ebiom.2026.106500 (PMC13656862; doi:10.1016/j.ebiom.2026.106500)
Supplement: Supplementary file 1 [file mmc1.docx]

**Table S1. Maternal and Infant Characteristics of Participants with Mtb-CMI Data at 6 Weeks of Life**

| **Variable** | **HEU** N = 23*^1^* | **HUU** N = 21*^1^* | **p-value***^2^* |
| --- | --- | --- | --- |
| **Sex (Male)** | 13 (57%) | 13 (62%) | >0.9 |
| **Vaginal Delivery** | 23 (100%) | 21 (100%) |  |
| **Gestational Age** | 39.00 (38.00, 40.00) | 39.00 (39.00, 40.00) | 0.6 |
| Missing | 2 | 0 |  |
| **Gestational Age >=37 weeks** | 21 (100%) | 20 (95%) | >0.9 |
| Missing | 2 | 0 |  |
| **Birth Weight (g)** | 3,150 (2,870, 3,450) | 3,425 (3,090, 3,645) | 0.12 |
| **Birth Weight <2500g** | 1 (4.3%) | 0 (0%) | >0.9 |
| *^1^*n (%); Median (Q1, Q3) | | | |
| *^2^*Pearson's Chi-squared test; Wilcoxon rank sum test | | | |

**Table S2. Correlation analyses of Mtb-specific IFNy and IL-2 responses at 6 weeks of life with maternal CD4 cell numbers and HIV RNA copies/ml**

| **Response** | **Predictor** | **rho** | **p_value** | **FDR_p** |
| --- | --- | --- | --- | --- |
| Mtb IFNγ | Maternal CD4 | 0.25 | 0.28 | 0.56 |
| Mtb IFNγ | Maternal HIV RNA | 0.12 | 0.59 | 0.78 |
| Mtb IL-2 | Maternal CD4 | 0.34 | 0.14 | 0.54 |
| Mtb IL-2 | Maternal HIV RNA | -0.06 | 0.79 | 0.79 |

*Spearman rank correlation

**Table S3. Demographic Characteristics of Infants with Measles and Tetanus CMI Data**

| **Variable** | **HEU** N = 29*^1^* | **HUU** N = 28*^1^* | **p-value***^2^* |
| --- | --- | --- | --- |
| **Sex (Male)** | 18 (62%) | 17 (61%) | >0.9 |
| **Vaginal Delivery** | 27 (93%) | 27 (96%) | >0.9 |
| **Gestational Age** | 39.00 (38.00, 40.00) | 40.00 (38.00, 40.00) | 0.2 |
| Missing | 2 | 0 |  |
| **Gestational Age >=37 weeks** | 27 (100%) | 28 (100%) |  |
| Missing | 2 | 0 |  |
| **Birth Weight (g)** | 3,110 (2,885, 3,195) | 3,195 (2,890, 3,673) | 0.2 |
| **Birth Weight <2500g** | 3 (10%) | 3 (11%) | >0.9 |
| *^1^*n (%); Median (Q1, Q3) | | | |
| *^2^*Pearson's Chi-squared test; Wilcoxon rank sum test | | | |

**Table S4. Demographic Characteristics of Infants with Measles Antibody Data**

| **Variable** | **HEU** N = 66*^1^* | **HUU** N = 68*^1^* | **p-value***^2^* |
| --- | --- | --- | --- |
| **Sex (Male)** | 31 (47%) | 41 (60%) | 0.2 |
| **Vaginal Delivery** | 62 (94%) | 67 (99%) | 0.3 |
| **Gestational Age** | 39.00 (38.00, 40.00) | 39.00 (38.00, 40.00) | >0.9 |
| Missing | 3 | 0 |  |
| **Gestational Age >37 weeks** | 62 (98%) | 66 (97%) | >0.9 |
| Missing | 3 | 0 |  |
| **Birth Weight (g)** | 3,100 (2,855, 3,285) | 3,263 (2,975, 3,553) | 0.014 |
| **Birth Weight <2500g** | 5 (7.6%) | 4 (5.9%) | >0.9 |
| *^1^*n (%); Median (Q1, Q3) | | | |
| *^2^*Pearson's Chi-squared test; Wilcoxon rank sum test | | | |

A

B

C

**Figure S1. Gating Strategies. Panels A and B** show gating trees used in Treg and Tici Panels 1 and 2, respectively.

**Panel C** shows fluorescence minus one (FMO) used to guide the gating of Treg and Tici markers

A. **Mtb IL-2**

**B. Mtb IFNγ**

**C. Tetanus IL-2**

**D. Tetanus IFNγ**

**E. Measles IL-2**

**F. Measles Antibodies**

**Figure S2. The effect of the gut microbiome on responses to vaccines in the first 62 weeks of life in HEU and HUU infants.** The graphs show the enrichment analysis for the outcome measure indicated in the panel title. Enrichment analyses included phyla with ≥10 genera in the study participants. Green continuous lines show the enrichment scores. Black vertical line represent the genera in each phylum. **Panels A and B** used data on responses to BCG measured at 6 weeks of life in 23 HEUs and 20 HUUs with paired maternal gut microbiome data. **Panels C and D** used data on responses to tetanus vaccine measured at 62 weeks of life in 27 HEUs and 27 HUUs with paired gut microbiome data at 28 weeks of life (closest study visit to the last dose of tetanus vaccine). **Panel E** used data from 26 HEUs and 27 HUUs with paired measles CMI and gut microbiome data at 62 weeks of life (the last dose of measles vaccine was administered at 52 weeks of life). **Panel F** used data from 59 HEUs and 69 HUUs with paired measles antibody and gut microbiome information.
